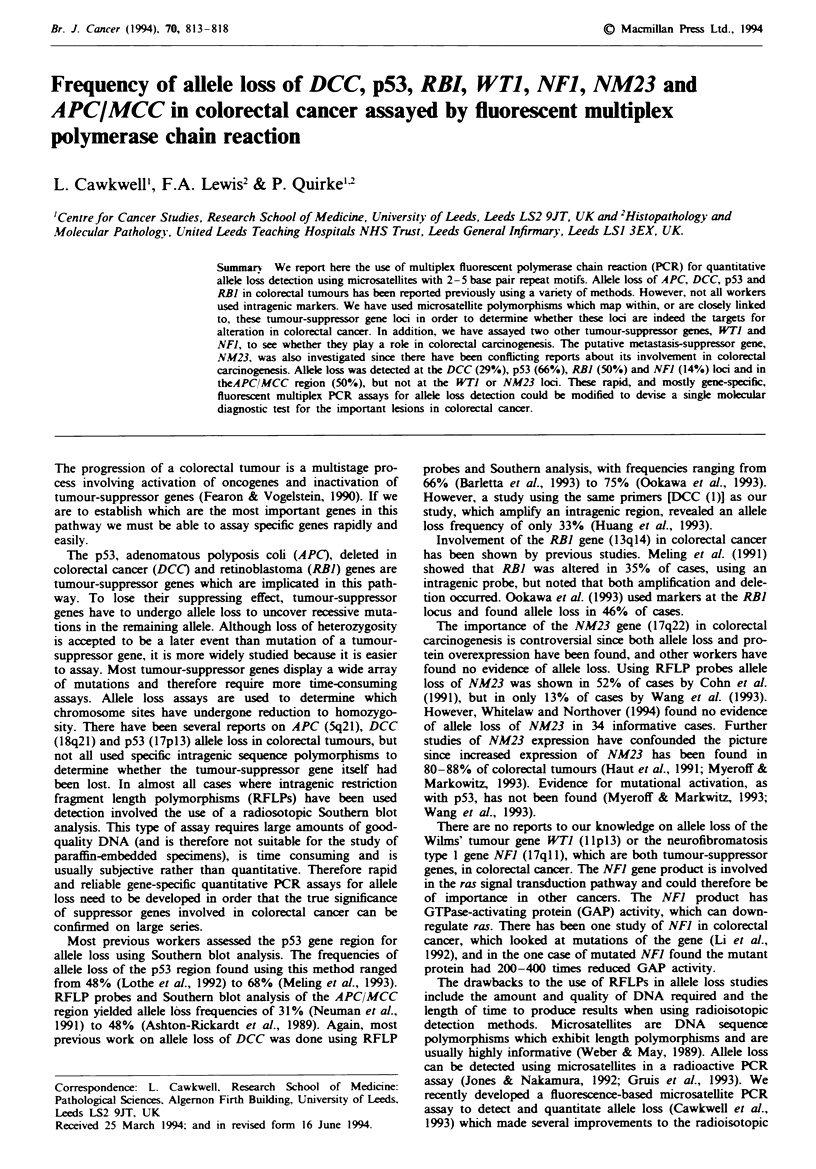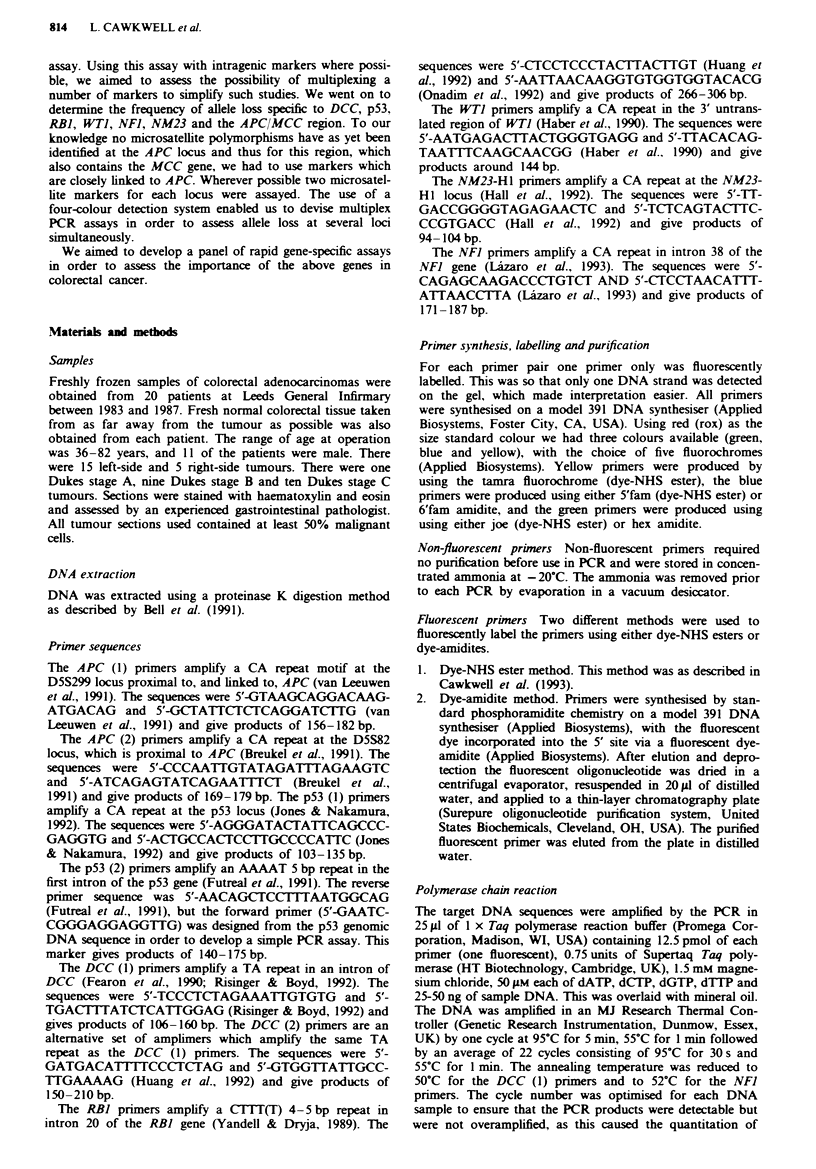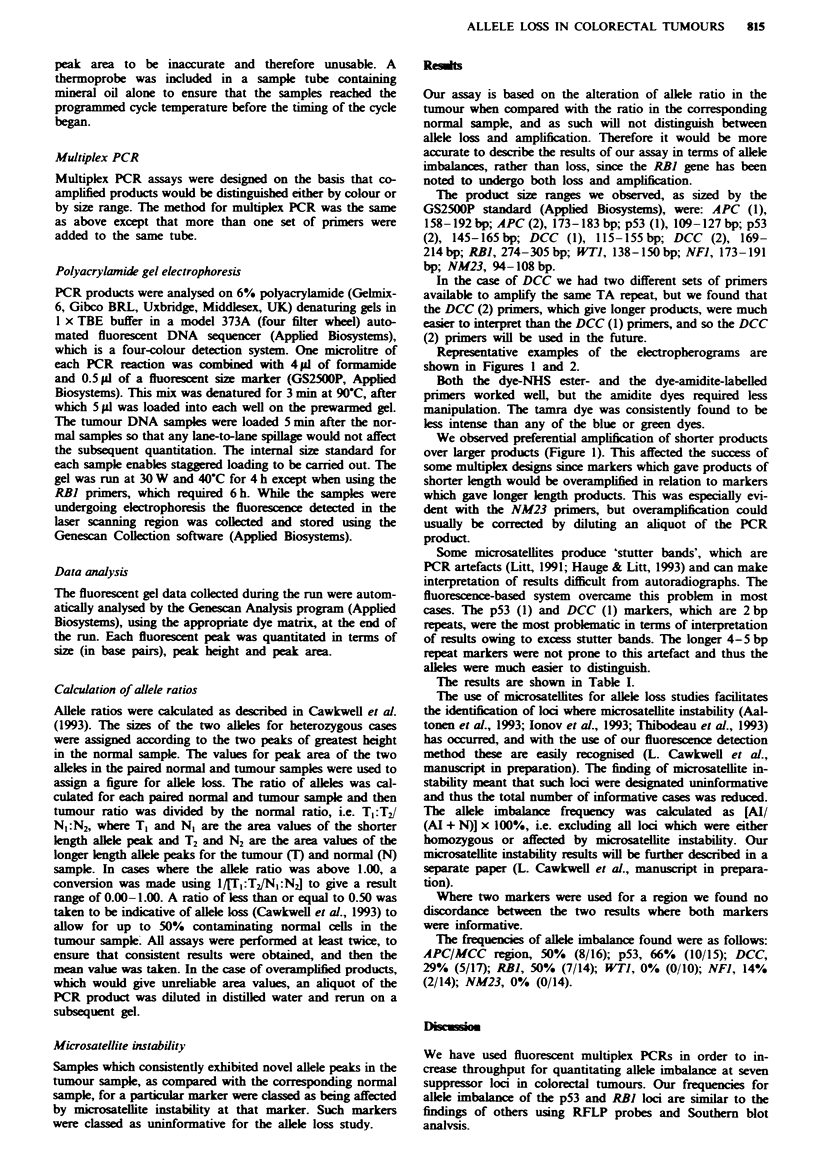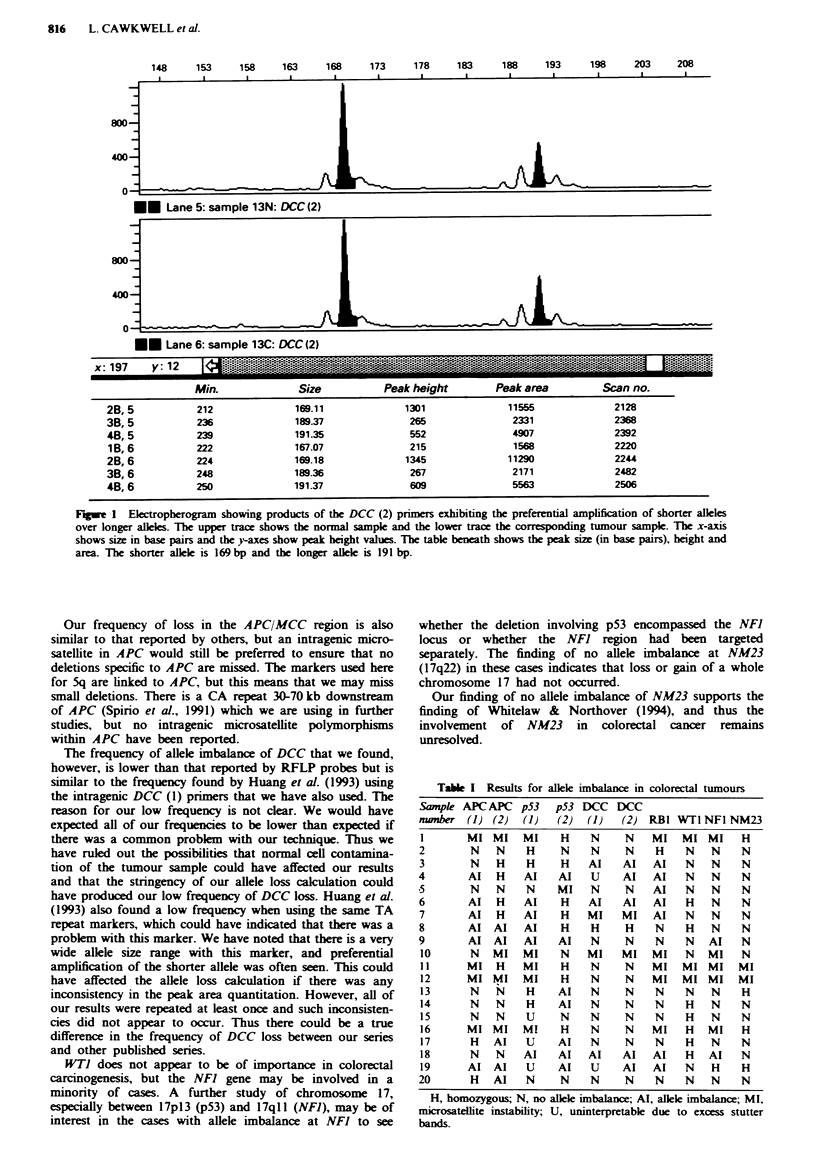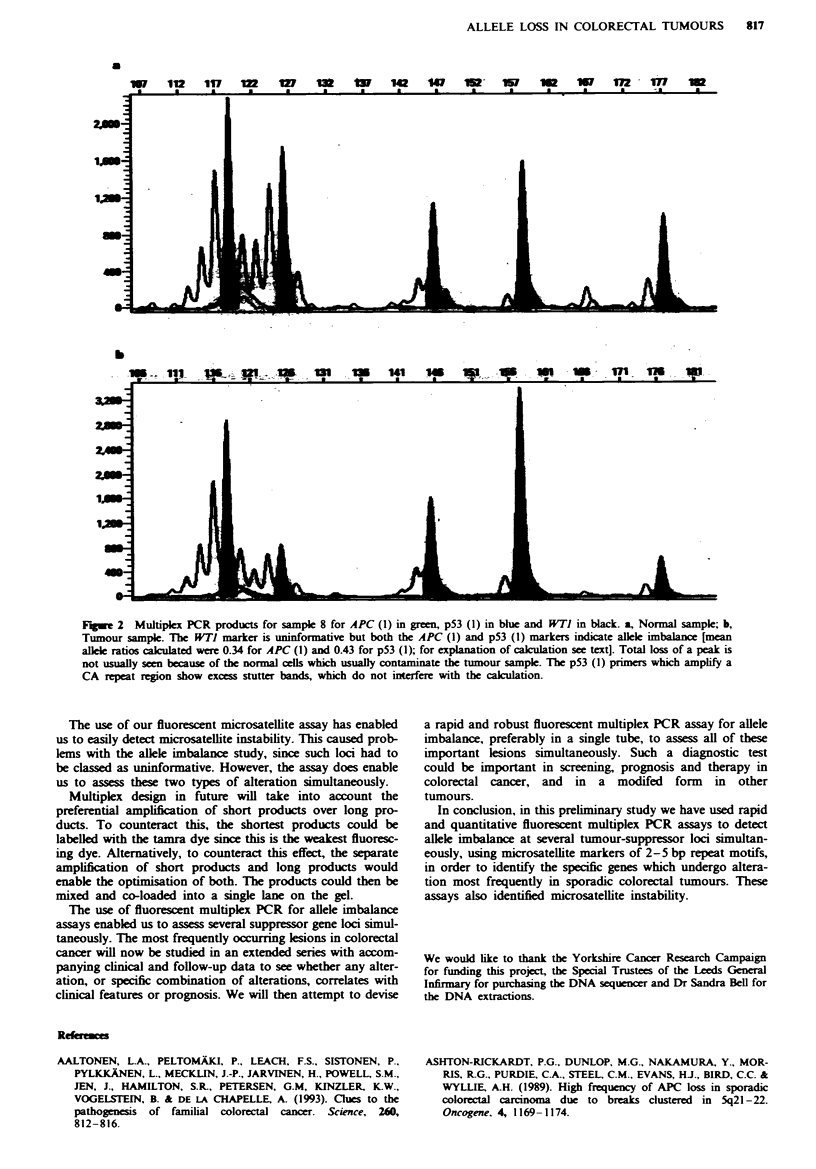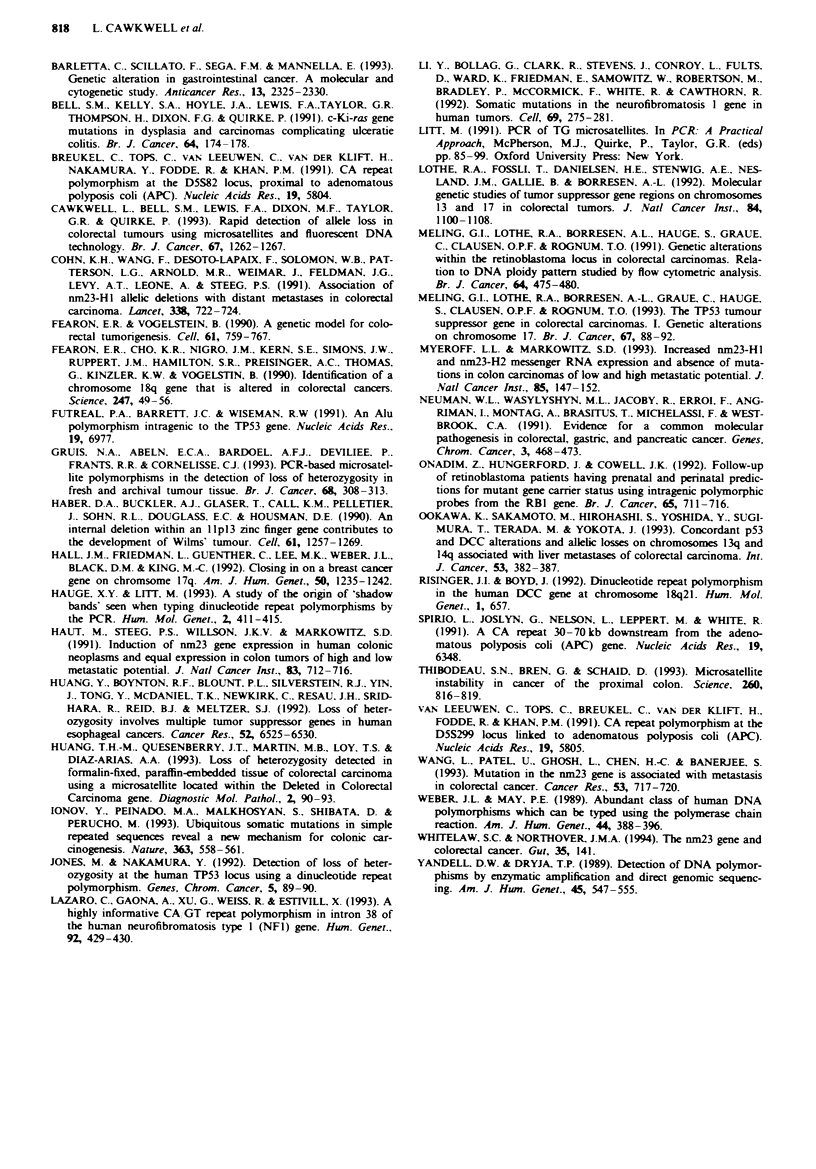# Frequency of allele loss of DCC, p53, RBI, WT1, NF1, NM23 and APC/MCC in colorectal cancer assayed by fluorescent multiplex polymerase chain reaction.

**DOI:** 10.1038/bjc.1994.404

**Published:** 1994-11

**Authors:** L. Cawkwell, F. A. Lewis, P. Quirke

**Affiliations:** Centre for Cancer Studies, Research School of Medicine, University of Leeds, UK.

## Abstract

**Images:**


Br. J. Cancer (1994), 70, 813-818 © Macmillan Press Ltd., 1994

### Frequency of allele loss of *DCC*, p53, *RBI*, *WT1*, *NF1*, *NM23* and *APC/MCC* in colorectal cancer assayed by fluorescent multiplex polymerase chain reaction

L. Cawkwell[1], F.A. Lewis[2] & P. Quirke[1,2]

[1]*Centre for Cancer Studies, Research School of Medicine, University of Leeds, Leeds LS2 9JT, UK and* [2]*Histopathology and Molecular Pathology, United Leeds Teaching Hospitals NHS Trust, Leeds General Infirmary, Leeds LS1 3EX, UK.*

**Summary** We report here the use of multiplex fluorescent polymerase chain reaction (PCR) for quantitative allele loss detection using microsatellites with 2-5 base pair repeat motifs. Allele loss of *APC*, *DCC*, p53 and *RB1* in colorectal tumours has been reported previously using a variety of methods. However, not all workers used intragenic markers. We have used microsatellite polymorphisms which map within, or are closely linked to, these tumour-suppressor gene loci in order to determine whether these loci are indeed the targets for alteration in colorectal cancer. In addition, we have assayed two other tumour-suppressor genes, *WT1* and *NF1*, to see whether they play a role in colorectal carcinogenesis. The putative metastasis-suppressor gene, *NM23*, was also investigated since there have been conflicting reports about its involvement in colorectal carcinogenesis. Allele loss was detected at the *DCC* (29%), p53 (66%), *RB1* (50%) and *NF1* (14%) loci and in the*APC/MCC* region (50%), but not at the *WT1* or *NM23* loci. These rapid, and mostly gene-specific, fluorescent multiplex PCR assays for allele loss detection could be modified to devise a single molecular diagnostic test for the important lesions in colorectal cancer.

The progression of a colorectal tumour is a multistage process involving activation of oncogenes and inactivation of tumour-suppressor genes (Fearon & Vogelstein, 1990). If we are to establish which are the most important genes in this pathway we must be able to assay specific genes rapidly and easily.

The p53, adenomatous polyposis coli (*APC*), deleted in colorectal cancer (*DCC*) and retinoblastoma (*RB1*) genes are tumour-suppressor genes which are implicated in this pathway. To lose their suppressing effect, tumour-suppressor genes have to undergo allele loss to uncover recessive mutations in the remaining allele. Although loss of heterozygosity is accepted to be a later event than mutation of a tumour-suppressor gene, it is more widely studied because it is easier to assay. Most tumour-suppressor genes display a wide array of mutations and therefore require more time-consuming assays. Allele loss assays are used to determine which chromosome sites have undergone reduction to homozygosity. There have been several reports on *APC* (5q21), *DCC* (18q21) and p53 (17p13) allele loss in colorectal tumours, but not all used specific intragenic sequence polymorphisms to determine whether the tumour-suppressor gene itself had been lost. In almost all cases where intragenic restriction fragment length polymorphisms (RFLPs) have been used detection involved the use of a radiosotopic Southern blot analysis. This type of assay requires large amounts of good-quality DNA (and is therefore not suitable for the study of paraffin-embedded specimens), is time consuming and is usually subjective rather than quantitative. Therefore rapid and reliable gene-specific quantitative PCR assays for allele loss need to be developed in order that the true significance of suppressor genes involved in colorectal cancer can be confirmed on large series.

Most previous workers assessed the p53 gene region for allele loss using Southern blot analysis. The frequencies of allele loss of the p53 region found using this method ranged from 48% (Lothe *et al.*, 1992) to 68% (Meling *et al.*, 1993). RFLP probes and Southern blot analysis of the *APC/MCC* region yielded allele lòss frequencies of 31% (Neuman *et al.*, 1991) to 48% (Ashton-Rickardt *et al.*, 1989). Again, most previous work on allele loss of *DCC* was done using RFLP probes and Southern analysis, with frequencies ranging from 66% (Barletta *et al.*, 1993) to 75% (Ookawa *et al.*, 1993). However, a study using the same primers [DCC (1)] as our study, which amplify an intragenic region, revealed an allele loss frequency of only 33% (Huang *et al.*, 1993).

Involvement of the *RB1* gene (13q14) in colorectal cancer has been shown by previous studies. Meling *et al.* (1991) showed that *RB1* was altered in 35% of cases, using an intragenic probe, but noted that both amplification and deletion occurred. Ookawa *et al.* (1993) used markers at the *RB1* locus and found allele loss in 46% of cases.

The importance of the *NM23* gene (17q22) in colorectal carcinogenesis is controversial since both allele loss and protein overexpression have been found, and other workers have found no evidence of allele loss. Using RFLP probes allele loss of *NM23* was shown in 52% of cases by Cohn *et al.* (1991), but in only 13% of cases by Wang *et al.* (1993). However, Whitelaw and Northover (1994) found no evidence of allele loss of *NM23* in 34 informative cases. Further studies of *NM23* expression have confounded the picture since increased expression of *NM23* has been found in 80-88% of colorectal tumours (Haut *et al.*, 1991; Myeroff & Markowitz, 1993). Evidence for mutational activation, as with p53, has not been found (Myeroff & Markwitz, 1993; Wang *et al.*, 1993).

There are no reports to our knowledge on allele loss of the Wilms' tumour gene *WT1* (11p13) or the neurofibromatosis type 1 gene *NF1* (17q11), which are both tumour-suppressor genes, in colorectal cancer. The *NF1* gene product is involved in the *ras* signal transduction pathway and could therefore be of importance in other cancers. The *NF1* product has GTPase-activating protein (GAP) activity, which can down-regulate *ras*. There has been one study of *NF1* in colorectal cancer, which looked at mutations of the gene (Li *et al.*, 1992), and in the one case of mutated *NF1* found the mutant protein had 200-400 times reduced GAP activity.

The drawbacks to the use of RFLPs in allele loss studies include the amount and quality of DNA required and the length of time to produce results when using radioisotopic detection methods. Microsatellites are DNA sequence polymorphisms which exhibit length polymorphisms and are usually highly informative (Weber & May, 1989). Allele loss can be detected using microsatellites in a radioactive PCR assay (Jones & Nakamura, 1992; Gruis *et al.*, 1993). We recently developed a fluorescence-based microsatellite PCR assay to detect and quantitate allele loss (Cawkwell *et al.*, 1993) which made several improvements to the radioisotopic

Correspondence: L. Cawkwell, Research School of Medicine: Pathological Sciences, Algernon Firth Building, University of Leeds, Leeds LS2 9JT, UK
Received 25 March 1994; and in revised form 16 June 1994.

assay. Using this assay with intragenic markers where possible, we aimed to assess the possibility of multiplexing a number of markers to simplify such studies. We went on to determine the frequency of allele loss specific to *DCC*, p53, *RB1*, *WT1*, *NF1*, *NM23* and the *APC/MCC* region. To our knowledge no microsatellite polymorphisms have as yet been identified at the *APC* locus and thus for this region, which also contains the *MCC* gene, we had to use markers which are closely linked to *APC*. Wherever possible two microsatellite markers for each locus were assayed. The use of a four-colour detection system enabled us to devise multiplex PCR assays in order to assess allele loss at several loci simultaneously.

We aimed to develop a panel of rapid gene-specific assays in order to assess the importance of the above genes in colorectal cancer.

### Materials and methods

### Samples

Freshly frozen samples of colorectal adenocarcinomas were obtained from 20 patients at Leeds General Infirmary between 1983 and 1987. Fresh normal colorectal tissue taken from as far away from the tumour as possible was also obtained from each patient. The range of age at operation was 36–82 years, and 11 of the patients were male. There were 15 left-side and 5 right-side tumours. There were one Dukes stage A, nine Dukes stage B and ten Dukes stage C tumours. Sections were stained with haematoxylin and eosin and assessed by an experienced gastrointestinal pathologist. All tumour sections used contained at least 50% malignant cells.

### DNA extraction

DNA was extracted using a proteinase K digestion method as described by Bell *et al.* (1991).

### Primer sequences

The *APC* (1) primers amplify a CA repeat motif at the D5S299 locus proximal to, and linked to, *APC* (van Leeuwen *et al.*, 1991). The sequences were 5′-GTAAGCAGGACAAG-ATGACAG and 5′-GCTATTCTCTCAGGATCTTG (van Leeuwen *et al.*, 1991) and give products of 156–182 bp.

The *APC* (2) primers amplify a CA repeat at the D5S82 locus, which is proximal to *APC* (Breukel *et al.*, 1991). The sequences were 5′-CCCAATTGTATAGATTTAGAAGTC and 5′-ATCAGAGTATCAGAATTTCT (Breukel *et al.*, 1991) and give products of 169–179 bp. The p53 (1) primers amplify a CA repeat at the p53 locus (Jones & Nakamura, 1992). The sequences were 5′-AGGGATACTATTCAGCCC-GAGGTG and 5′-ACTGCCACTCCTTGCCCCATTC (Jones & Nakamura, 1992) and give products of 103–135 bp.

The p53 (2) primers amplify an AAAAT 5 bp repeat in the first intron of the p53 gene (Futreal *et al.*, 1991). The reverse primer sequence was 5′-AACAGCTCCTTTAATGGCAG (Futreal *et al.*, 1991), but the forward primer (5′-GAATC-CGGGAGGAGGTTG) was designed from the p53 genomic DNA sequence in order to develop a simple PCR assay. This marker gives products of 140–175 bp.

The *DCC* (1) primers amplify a TA repeat in an intron of *DCC* (Fearon *et al.*, 1990; Risinger & Boyd, 1992). The sequences were 5′-TCCCTCTAGAAATTGTGTG and 5′-TGACTTTATCTCATTGGAG (Risinger & Boyd, 1992) and gives products of 106–160 bp. The *DCC* (2) primers are an alternative set of amplimers which amplify the same TA repeat as the *DCC* (1) primers. The sequences were 5′-GATGACATTTTCCCTCTAG and 5′-GTGGTTATTGCC-TTGAAAAG (Huang *et al.*, 1992) and give products of 150–210 bp.

The *RB1* primers amplify a CTTT(T) 4–5 bp repeat in intron 20 of the *RB1* gene (Yandell & Dryja, 1989). The sequences were 5′-CTCCTCCCTACTTACTTGT (Huang *et al.*, 1992) and 5′-AATTAACAAGGTGTGGTGGTACACG (Onadim *et al.*, 1992) and give products of 266–306 bp.

The *WT1* primers amplify a CA repeat in the 3′ untranslated region of *WT1* (Haber *et al.*, 1990). The sequences were 5′-AATGAGACTTACTGGGTGAGG and 5′-TTACACAG-TAATTTCAAGCAACGG (Haber *et al.*, 1990) and give products around 144 bp.

The *NM23*-H1 primers amplify a CA repeat at the *NM23*-H1 locus (Hall *et al.*, 1992). The sequences were 5′-TT-GACCGGGGTAGAGAACTC and 5′-TCTCAGTACTTC-CCGTGACC (Hall *et al.*, 1992) and give products of 94–104 bp.

The *NF1* primers amplify a CA repeat in intron 38 of the *NF1* gene (Lázaro *et al.*, 1993). The sequences were 5′-CAGAGCAAGACCCTGTCT AND 5′-CTCCTAACATTT-ATTAACCTTA (Lázaro *et al.*, 1993) and give products of 171–187 bp.

### Primer synthesis, labelling and purification

For each primer pair one primer only was fluorescently labelled. This was so that only one DNA strand was detected on the gel, which made interpretation easier. All primers were synthesised on a model 391 DNA synthesiser (Applied Biosystems, Foster City, CA, USA). Using red (rox) as the size standard colour we had three colours available (green, blue and yellow), with the choice of five fluorochromes (Applied Biosystems). Yellow primers were produced by using the tamra fluorochrome (dye-NHS ester), the blue primers were produced using either 5′fam (dye-NHS ester) or 6′fam amidite, and the green primers were produced using using either joe (dye-NHS ester) or hex amidite.

*Non-fluorescent primers* Non-fluorescent primers required no purification before use in PCR and were stored in concentrated ammonia at −20°C. The ammonia was removed prior to each PCR by evaporation in a vacuum desiccator.

*Fluorescent primers* Two different methods were used to fluorescently label the primers using either dye-NHS esters or dye-amidites.

1. Dye-NHS ester method. This method was as described in Cawkwell *et al.* (1993).
2. Dye-amidite method. Primers were synthesised by standard phosphoramidite chemistry on a model 391 DNA synthesiser (Applied Biosystems), with the fluorescent dye incorporated into the 5′ site via a fluorescent dye-amidite (Applied Biosystems). After elution and deprotection the fluorescent oligonucleotide was dried in a centrifugal evaporator, resuspended in 20 µl of distilled water, and applied to a thin-layer chromatography plate (Surepure oligonucleotide purification system, United States Biochemicals, Cleveland, OH, USA). The purified fluorescent primer was eluted from the plate in distilled water.

### Polymerase chain reaction

The target DNA sequences were amplified by the PCR in 25 µl of 1 × *Taq* polymerase reaction buffer (Promega Corporation, Madison, WI, USA) containing 12.5 pmol of each primer (one fluorescent), 0.75 units of Supertaq *Taq* polymerase (HT Biotechnology, Cambridge, UK), 1.5 mM magnesium chloride, 50 µM each of dATP, dCTP, dGTP, dTTP and 25-50 ng of sample DNA. This was overlaid with mineral oil. The DNA was amplified in an MJ Research Thermal Controller (Genetic Research Instrumentation, Dunmow, Essex, UK) by one cycle at 95°C for 5 min, 55°C for 1 min followed by an average of 22 cycles consisting of 95°C for 30 s and 55°C for 1 min. The annealing temperature was reduced to 50°C for the *DCC* (1) primers and to 52°C for the *NF1* primers. The cycle number was optimised for each DNA sample to ensure that the PCR products were detectable but were not overamplified, as this caused the quantitation of peak area to be inaccurate and therefore unusable. A thermoprobe was included in a sample tube containing mineral oil alone to ensure that the samples reached the programmed cycle temperature before the timing of the cycle began.

### *Multiplex PCR*

Multiplex PCR assays were designed on the basis that co-amplified products would be distinguished either by colour or by size range. The method for multiplex PCR was the same as above except that more than one set of primers were added to the same tube.

### *Polyacrylamide gel electrophoresis*

PCR products were analysed on 6% polyacrylamide (Gelmix-6, Gibco BRL, Uxbridge, Middlesex, UK) denaturing gels in 1 × TBE buffer in a model 373A (four filter wheel) automated fluorescent DNA sequencer (Applied Biosystems), which is a four-colour detection system. One microlitre of each PCR reaction was combined with 4 µl of formamide and 0.5 µl of a fluorescent size marker (GS2500P, Applied Biosystems). This mix was denatured for 3 min at 90°C, after which 5 µl was loaded into each well on the prewarmed gel. The tumour DNA samples were loaded 5 min after the normal samples so that any lane-to-lane spillage would not affect the subsequent quantitation. The internal size standard for each sample enables staggered loading to be carried out. The gel was run at 30 W and 40°C for 4 h except when using the *RB1* primers, which required 6 h. While the samples were undergoing electrophoresis the fluorescence detected in the laser scanning region was collected and stored using the Genescan Collection software (Applied Biosystems).

### *Data analysis*

The fluorescent gel data collected during the run were automatically analysed by the Genescan Analysis program (Applied Biosystems), using the appropriate dye matrix, at the end of the run. Each fluorescent peak was quantitated in terms of size (in base pairs), peak height and peak area.

### *Calculation of allele ratios*

Allele ratios were calculated as described in Cawkwell *et al.* (1993). The sizes of the two alleles for heterozygous cases were assigned according to the two peaks of greatest height in the normal sample. The values for peak area of the two alleles in the paired normal and tumour samples were used to assign a figure for allele loss. The ratio of alleles was calculated for each paired normal and tumour sample and then tumour ratio was divided by the normal ratio, i.e. $T_1:T_2/N_1:N_2$, where $T_1$ and $N_1$ are the area values of the shorter length allele peak and $T_2$ and $N_2$ are the area values of the longer length allele peaks for the tumour (T) and normal (N) sample. In cases where the allele ratio was above 1.00, a conversion was made using $1/[T_1:T_2/N_1:N_2]$ to give a result range of 0.00–1.00. A ratio of less than or equal to 0.50 was taken to be indicative of allele loss (Cawkwell *et al.*, 1993) to allow for up to 50% contaminating normal cells in the tumour sample. All assays were performed at least twice, to ensure that consistent results were obtained, and then the mean value was taken. In the case of overamplified products, which would give unreliable area values, an aliquot of the PCR product was diluted in distilled water and rerun on a subsequent gel.

### *Microsatellite instability*

Samples which consistently exhibited novel allele peaks in the tumour sample, as compared with the corresponding normal sample, for a particular marker were classed as being affected by microsatellite instability at that marker. Such markers were classed as uninformative for the allele loss study.

### Results

Our assay is based on the alteration of allele ratio in the tumour when compared with the ratio in the corresponding normal sample, and as such will not distinguish between allele loss and amplification. Therefore it would be more accurate to describe the results of our assay in terms of allele imbalances, rather than loss, since the *RB1* gene has been noted to undergo both loss and amplification.

The product size ranges we observed, as sized by the GS2500P standard (Applied Biosystems), were: *APC* (1), 158–192 bp; *APC* (2), 173–183 bp; p53 (1), 109–127 bp; p53 (2), 145–165 bp; *DCC* (1), 115–155 bp; *DCC* (2), 169–214 bp; *RB1*, 274–305 bp; *WT1*, 138–150 bp; *NF1*, 173–191 bp; *NM23*, 94–108 bp.

In the case of *DCC* we had two different sets of primers available to amplify the same TA repeat, but we found that the *DCC* (2) primers, which give longer products, were much easier to interpret than the *DCC* (1) primers, and so the *DCC* (2) primers will be used in the future.

Representative examples of the electropherograms are shown in Figures 1 and 2.

Both the dye-NHS ester- and the dye-amidite-labelled primers worked well, but the amidite dyes required less manipulation. The tamra dye was consistently found to be less intense than any of the blue or green dyes.

We observed preferential amplification of shorter products over larger products (Figure 1). This affected the success of some multiplex designs since markers which gave products of shorter length would be overamplified in relation to markers which gave longer length products. This was especially evident with the *NM23* primers, but overamplification could usually be corrected by diluting an aliquot of the PCR product.

Some microsatellites produce 'stutter bands', which are PCR artefacts (Litt, 1991; Hauge & Litt, 1993) and can make interpretation of results difficult from autoradiographs. The fluorescence-based system overcame this problem in most cases. The p53 (1) and *DCC* (1) markers, which are 2 bp repeats, were the most problematic in terms of interpretation of results owing to excess stutter bands. The longer 4–5 bp repeat markers were not prone to this artefact and thus the alleles were much easier to distinguish.

The results are shown in Table I.

The use of microsatellites for allele loss studies facilitates the identification of loci where microsatellite instability (Aaltonen *et al.*, 1993; Ionov *et al.*, 1993; Thibodeau *et al.*, 1993) has occurred, and with the use of our fluorescence detection method these are easily recognised (L. Cawkwell *et al.*, manuscript in preparation). The finding of microsatellite instability meant that such loci were designated uninformative and thus the total number of informative cases was reduced. The allele imbalance frequency was calculated as [AI/(AI + N)] × 100%, i.e. excluding all loci which were either homozygous or affected by microsatellite instability. Our microsatellite instability results will be further described in a separate paper (L. Cawkwell *et al.*, manuscript in preparation).

Where two markers were used for a region we found no discordance between the two results where both markers were informative.

The frequencies of allele imbalance found were as follows: *APC/MCC* region, 50% (8/16); p53, 66% (10/15); *DCC*, 29% (5/17); *RB1*, 50% (7/14); *WT1*, 0% (0/10); *NF1*, 14% (2/14); *NM23*, 0% (0/14).

### Discussion

We have used fluorescent multiplex PCRs in order to increase throughput for quantitating allele imbalance at seven suppressor loci in colorectal tumours. Our frequencies for allele imbalance of the p53 and *RB1* loci are similar to the findings of others using RFLP probes and Southern blot analysis.

| | Min. | Size | Peak height | Peak area | Scan no. |
|---|---|---|---|---|---|
| 2B, 5 | 212 | 169.11 | 1301 | 11555 | 2128 |
| 3B, 5 | 236 | 189.37 | 265 | 2331 | 2368 |
| 4B, 5 | 239 | 191.35 | 552 | 4907 | 2392 |
| 1B, 6 | 222 | 167.07 | 215 | 1568 | 2220 |
| 2B, 6 | 224 | 169.18 | 1345 | 11290 | 2244 |
| 3B, 6 | 248 | 189.36 | 267 | 2171 | 2482 |
| 4B, 6 | 250 | 191.37 | 609 | 5563 | 2506 |

**Figure 1** Electropherogram showing products of the *DCC* (2) primers exhibiting the preferential amplification of shorter alleles over longer alleles. The upper trace shows the normal sample and the lower trace the corresponding tumour sample. The *x*-axis shows size in base pairs and the *y*-axes show peak height values. The table beneath shows the peak size (in base pairs), height and area. The shorter allele is 169 bp and the longer allele is 191 bp.

Our frequency of loss in the *APC/MCC* region is also similar to that reported by others, but an intragenic microsatellite in *APC* would still be preferred to ensure that no deletions specific to *APC* are missed. The markers used here for 5q are linked to *APC*, but this means that we may miss small deletions. There is a CA repeat 30-70 kb downstream of *APC* (Spirio *et al*., 1991) which we are using in further studies, but no intragenic microsatellite polymorphisms within *APC* have been reported.

The frequency of allele imbalance of *DCC* that we found, however, is lower than that reported by RFLP probes but is similar to the frequency found by Huang *et al*. (1993) using the intragenic *DCC* (1) primers that we have also used. The reason for our low frequency is not clear. We would have expected all of our frequencies to be lower than expected if there was a common problem with our technique. Thus we have ruled out the possibilities that normal cell contamination of the tumour sample could have affected our results and that the stringency of our allele loss calculation could have produced our low frequency of *DCC* loss. Huang *et al*. (1993) also found a low frequency when using the same TA repeat markers, which could have indicated that there was a problem with this marker. We have noted that there is a very wide allele size range with this marker, and preferential amplification of the shorter allele was often seen. This could have affected the allele loss calculation if there was any inconsistency in the peak area quantitation. However, all of our results were repeated at least once and such inconsistencies did not appear to occur. Thus there could be a true difference in the frequency of *DCC* loss between our series and other published series.

*WT1* does not appear to be of importance in colorectal carcinogenesis, but the *NF1* gene may be involved in a minority of cases. A further study of chromosome 17, especially between 17p13 (p53) and 17q11 (*NF1*), may be of interest in the cases with allele imbalance at *NF1* to see whether the deletion involving p53 encompassed the *NF1* locus or whether the *NF1* region had been targeted separately. The finding of no allele imbalance at *NM23* (17q22) in these cases indicates that loss or gain of a whole chromosome 17 had not occurred.

Our finding of no allele imbalance of *NM23* supports the finding of Whitelaw & Northover (1994), and thus the involvement of *NM23* in colorectal cancer remains unresolved.

**Table I** Results for allele imbalance in colorectal tumours

| *Sample number* | APC *(1)* | APC *(2)* | *p53 (1)* | *p53 (2)* | DCC *(1)* | DCC *(2)* | RB1 | WT1 | NF1 | NM23 |
|---|---|---|---|---|---|---|---|---|---|---|
| 1 | MI | MI | MI | H | N | N | MI | MI | MI | H |
| 2 | N | N | H | N | N | N | H | N | N | N |
| 3 | N | H | H | H | AI | AI | AI | N | N | N |
| 4 | AI | H | AI | AI | U | AI | AI | N | N | N |
| 5 | N | N | N | MI | N | N | AI | N | N | N |
| 6 | AI | H | AI | H | AI | AI | AI | H | N | N |
| 7 | AI | H | AI | H | MI | MI | AI | N | N | N |
| 8 | AI | AI | AI | H | H | H | N | H | N | N |
| 9 | AI | AI | AI | AI | N | N | N | N | AI | N |
| 10 | N | MI | MI | N | MI | MI | MI | N | MI | N |
| 11 | MI | H | MI | H | N | N | MI | MI | MI | MI |
| 12 | MI | MI | MI | H | N | N | MI | MI | MI | MI |
| 13 | N | N | H | AI | N | N | N | N | N | H |
| 14 | N | N | H | AI | N | N | N | H | N | N |
| 15 | N | N | U | N | N | N | N | H | N | N |
| 16 | MI | MI | MI | H | N | N | MI | H | MI | H |
| 17 | H | AI | U | AI | N | N | N | H | N | N |
| 18 | N | N | AI | AI | AI | AI | AI | H | AI | N |
| 19 | AI | AI | U | AI | U | AI | AI | N | H | H |
| 20 | H | AI | N | N | N | N | N | N | N | N |

H, homozygous; N, no allele imbalance; AI, allele imbalance; MI, microsatellite instability; U, uninterpretable due to excess stutter bands.

**Figure 2** Multiplex PCR products for sample 8 for *APC* (1) in green, p53 (1) in blue and *WT1* in black. **a**, Normal sample; **b**, Tumour sample. The *WT1* marker is uninformative but both the *APC* (1) and p53 (1) markers indicate allele imbalance [mean allele ratios calculated were 0.34 for *APC* (1) and 0.43 for p53 (1); for explanation of calculation see text]. Total loss of a peak is not usually seen because of the normal cells which usually contaminate the tumour sample. The p53 (1) primers which amplify a CA repeat region show excess stutter bands, which do not interfere with the calculation.

The use of our fluorescent microsatellite assay has enabled us to easily detect microsatellite instability. This caused problems with the allele imbalance study, since such loci had to be classed as uninformative. However, the assay does enable us to assess these two types of alteration simultaneously.

Multiplex design in future will take into account the preferential amplification of short products over long products. To counteract this, the shortest products could be labelled with the tamra dye since this is the weakest fluorescing dye. Alternatively, to counteract this effect, the separate amplification of short products and long products would enable the optimisation of both. The products could then be mixed and co-loaded into a single lane on the gel.

The use of fluorescent multiplex PCR for allele imbalance assays enabled us to assess several suppressor gene loci simultaneously. The most frequently occurring lesions in colorectal cancer will now be studied in an extended series with accompanying clinical and follow-up data to see whether any alteration, or specific combination of alterations, correlates with clinical features or prognosis. We will then attempt to devise a rapid and robust fluorescent multiplex PCR assay for allele imbalance, preferably in a single tube, to assess all of these important lesions simultaneously. Such a diagnostic test could be important in screening, prognosis and therapy in colorectal cancer, and in a modifed form in other tumours.

In conclusion, in this preliminary study we have used rapid and quantitative fluorescent multiplex PCR assays to detect allele imbalance at several tumour-suppressor loci simultaneously, using microsatellite markers of 2–5 bp repeat motifs, in order to identify the specific genes which undergo alteration most frequently in sporadic colorectal tumours. These assays also identified microsatellite instability.

We would like to thank the Yorkshire Cancer Research Campaign for funding this project, the Special Trustees of the Leeds General Infirmary for purchasing the DNA sequencer and Dr Sandra Bell for the DNA extractions.

### References

AALTONEN, L.A., PELTOMÄKI, P., LEACH, F.S., SISTONEN, P., PYLKKÄNEN, L., MECKLIN, J.-P., JARVINEN, H., POWELL, S.M., JEN, J., HAMILTON, S.R., PETERSEN, G.M, KINZLER, K.W., VOGELSTEIN, B. & DE LA CHAPELLE, A. (1993). Clues to the pathogenesis of familial colorectal cancer. *Science*, **260**, 812–816.

ASHTON-RICKARDT, P.G., DUNLOP, M.G., NAKAMURA, Y., MORRIS, R.G., PURDIE, C.A., STEEL, C.M., EVANS, H.J., BIRD, C.C. & WYLLIE, A.H. (1989). High frequency of APC loss in sporadic colorectal carcinoma due to breaks clustered in 5q21–22. *Oncogene*, **4**, 1169–1174.

BARLETTA, C., SCILLATO, F., SEGA, F.M. & MANNELLA, E. (1993). Genetic alteration in gastrointestinal cancer. A molecular and cytogenetic study. *Anticancer Res.*, **13**, 2325-2330.

BELL, S.M., KELLY, S.A., HOYLE, J.A., LEWIS, F.A.,TAYLOR, G.R. THOMPSON, H., DIXON, F.G. & QUIRKE, P. (1991). c-Ki-*ras* gene mutations in dysplasia and carcinomas complicating ulceratie colitis. *Br. J. Cancer*, **64**, 174-178.

BREUKEL, C., TOPS, C., VAN LEEUWEN, C., VAN DER KLIFT, H., NAKAMURA, Y., FODDE, R. & KHAN, P.M. (1991). CA repeat polymorphism at the D5S82 locus, proximal to adenomatous polyposis coli (APC). *Nucleic Acids Res.*, **19**, 5804.

CAWKWELL, L., BELL, S.M., LEWIS, F.A., DIXON, M.F., TAYLOR, G.R. & QUIRKE, P. (1993). Rapid detection of allele loss in colorectal tumours using microsatellites and fluorescent DNA technology. *Br. J. Cancer*, **67**, 1262-1267.

COHN, K.H., WANG, F., DESOTO-LAPAIX, F., SOLOMON, W.B., PATTERSON, L.G., ARNOLD, M.R., WEIMAR, J., FELDMAN, J.G., LEVY, A.T., LEONE, A. & STEEG, P.S. (1991). Association of nm23-H1 allelic deletions with distant metastases in colorectal carcinoma. *Lancet*, **338**, 722-724.

FEARON, E.R. & VOGELSTEIN, B. (1990). A genetic model for colorectal tumorigenesis. *Cell*, **61**, 759-767.

FEARON, E.R., CHO, K.R., NIGRO, J.M., KERN, S.E., SIMONS, J.W., RUPPERT, J.M., HAMILTON, S.R., PREISINGER, A.C., THOMAS, G., KINZLER, K.W. & VOGELSTIN, B. (1990). Identification of a chromosome 18q gene that is altered in colorectal cancers. *Science*, **247**, 49-56.

FUTREAL, P.A., BARRETT, J.C. & WISEMAN, R.W (1991). An Alu polymorphism intragenic to the TP53 gene. *Nucleic Acids Res.*, **19**, 6977.

GRUIS, N.A., ABELN, E.C.A., BARDOEL, A.F.J., DEVILIEE, P., FRANTS, R.R. & CORNELISSE, C.J. (1993). PCR-based microsatellite polymorphisms in the detection of loss of heterozygosity in fresh and archival tumour tissue. *Br. J. Cancer*, **68**, 308-313.

HABER, D.A., BUCKLER, A.J., GLASER, T., CALL, K.M., PELLETIER, J., SOHN, R.L., DOUGLASS, E.C. & HOUSMAN, D.E. (1990). An internal deletion within an 11p13 zinc finger gene contributes to the development of Wilms' tumour. *Cell*, **61**, 1257-1269.

HALL, J.M., FRIEDMAN, L., GUENTHER, C., LEE, M.K., WEBER, J.L., BLACK, D.M. & KING, M.-C. (1992). Closing in on a breast cancer gene on chromsome 17q. *Am. J. Hum. Genet.*, **50**, 1235-1242.

HAUGE, X.Y. & LITT, M. (1993). A study of the origin of 'shadow bands' seen when typing dinucleotide repeat polymorphisms by the PCR. *Hum. Mol. Genet.*, **2**, 411-415.

HAUT, M., STEEG, P.S., WILLSON, J.K.V. & MARKOWITZ, S.D. (1991). Induction of nm23 gene expression in human colonic neoplasms and equal expression in colon tumors of high and low metastatic potential. *J. Natl Cancer Inst.*, **83**, 712-716.

HUANG, Y., BOYNTON, R.F., BLOUNT, P.L., SILVERSTEIN, R.J., YIN, J., TONG, Y., McDANIEL, T.K., NEWKIRK, C., RESAU, J.H., SRIDHARA, R., REID, B.J. & MELTZER, S.J. (1992). Loss of heterozygosity involves multiple tumor suppressor genes in human esophageal cancers. *Cancer Res.*, **52**, 6525-6530.

HUANG, T.H.-M., QUESENBERRY, J.T., MARTIN, M.B., LOY, T.S. & DIAZ-ARIAS, A.A. (1993). Loss of heterozygosity detected in formalin-fixed, paraffin-embedded tissue of colorectal carcinoma using a microsatellite located within the Deleted in Colorectal Carcinoma gene. *Diagnostic Mol. Pathol.*, **2**, 90-93.

IONOV, Y., PEINADO, M.A., MALKHOSYAN, S., SHIBATA, D. & PERUCHO, M. (1993). Ubiquitous somatic mutations in simple repeated sequences reveal a new mechanism for colonic carcinogenesis. *Nature*, **363**, 558-561.

JONES, M. & NAKAMURA, Y. (1992). Detection of loss of heterozygosity at the human TP53 locus using a dinucleotide repeat polymorphism. *Genes, Chrom. Cancer*, **5**, 89-90.

LAZARO, C., GAONA, A., XU, G., WEISS, R. & ESTIVILL, X. (1993). A highly informative CA/GT repeat polymorphism in intron 38 of the human neurofibromatosis type 1 (NF1) gene. *Hum. Genet.*, **92**, 429-430.

LI, Y., BOLLAG, G., CLARK, R., STEVENS, J., CONROY, L., FULTS, D., WARD, K., FRIEDMAN, E., SAMOWITZ, W., ROBERTSON, M., BRADLEY, P., McCORMICK, F., WHITE, R. & CAWTHORN, R. (1992). Somatic mutations in the neurofibromatosis 1 gene in human tumors. *Cell*, **69**, 275-281.

LITT, M. (1991). PCR of TG microsatellites. In *PCR: A Practical Approach*, McPherson, M.J., Quirke, P., Taylor, G.R. (eds) pp. 85-99. Oxford University Press: New York.

LOTHE, R.A., FOSSLI, T., DANIELSEN, H.E., STENWIG, A.E., NESLAND, J.M., GALLIE, B. & BORRESEN, A.-L. (1992). Molecular genetic studies of tumor suppressor gene regions on chromosomes 13 and 17 in colorectal tumors. *J. Natl Cancer Inst.*, **84**, 1100-1108.

MELING, G.I., LOTHE, R.A., BORRESEN, A.L., HAUGE, S., GRAUE, C., CLAUSEN, O.P.F. & ROGNUM, T.O. (1991). Genetic alterations within the retinoblastoma locus in colorectal carcinomas. Relation to DNA ploidy pattern studied by flow cytometric analysis. *Br. J. Cancer*, **64**, 475-480.

MELING, G.I., LOTHE, R.A., BORRESEN, A.-L., GRAUE, C., HAUGE, S., CLAUSEN, O.P.F. & ROGNUM, T.O. (1993). The TP53 tumour suppressor gene in colorectal carcinomas. I. Genetic alterations on chromosome 17. *Br. J. Cancer*, **67**, 88-92.

MYEROFF, L.L. & MARKOWITZ, S.D. (1993). Increased nm23-H1 and nm23-H2 messenger RNA expression and absence of mutations in colon carcinomas of low and high metastatic potential. *J. Natl Cancer Inst.*, **85**, 147-152.

NEUMAN, W.L., WASYLYSHYN, M.L., JACOBY, R., ERROI, F., ANGRIMAN, I., MONTAG, A., BRASITUS, T., MICHELASSI, F. & WESTBROOK, C.A. (1991). Evidence for a common molecular pathogenesis in colorectal, gastric, and pancreatic cancer. *Genes, Chrom. Cancer*, **3**, 468-473.

ONADIM, Z., HUNGERFORD, J. & COWELL, J.K. (1992). Follow-up of retinoblastoma patients having prenatal and perinatal predictions for mutant gene carrier status using intragenic polymorphic probes from the RB1 gene. *Br. J. Cancer*, **65**, 711-716.

OOKAWA, K., SAKAMOTO, M., HIROHASHI, S., YOSHIDA, Y., SUGIMURA, T., TERADA, M. & YOKOTA, J. (1993). Concordant p53 and DCC alterations and allelic losses on chromosomes 13q and 14q associated with liver metastases of colorectal carcinoma. *Int. J. Cancer*, **53**, 382-387.

RISINGER, J.I. & BOYD, J. (1992). Dinucleotide repeat polymorphism in the human DCC gene at chromosome 18q21. *Hum. Mol. Genet.*, **1**, 657.

SPIRIO, L., JOSLYN, G., NELSON, L., LEPPERT, M. & WHITE, R. (1991). A CA repeat 30-70 kb downstream from the adenomatous polyposis coli (APC) gene. *Nucleic Acids Res.*, **19**, 6348.

THIBODEAU, S.N., BREN, G. & SCHAID, D. (1993). Microsatellite instability in cancer of the proximal colon. *Science*, **260**, 816-819.

VAN LEEUWEN, C., TOPS, C., BREUKEL, C., VAN DER KLIFT, H., FODDE, R. & KHAN, P.M. (1991). CA repeat polymorphism at the D5S299 locus linked to adenomatous polyposis coli (APC). *Nucleic Acids Res.*, **19**, 5805.

WANG, L., PATEL, U., GHOSH, L., CHEN, H.-C. & BANERJEE, S. (1993). Mutation in the nm23 gene is associated with metastasis in colorectal cancer. *Cancer Res.*, **53**, 717-720.

WEBER, J.L. & MAY, P.E. (1989). Abundant class of human DNA polymorphisms which can be typed using the polymerase chain reaction. *Am. J. Hum. Genet.*, **44**, 388-396.

WHITELAW, S.C. & NORTHOVER, J.M.A. (1994). The nm23 gene and colorectal cancer. *Gut*, **35**, 141.

YANDELL, D.W. & DRYJA, T.P. (1989). Detection of DNA polymorphisms by enzymatic amplification and direct genomic sequencing. *Am. J. Hum. Genet.*, **45**, 547-555.